# Comparison of the modified Collard and hand‐sewn anastomosis for cervical esophagogastric anastomosis after esophagectomy in esophageal cancer patients: A propensity score‐matched analysis

**DOI:** 10.1002/ags3.12220

**Published:** 2018-11-08

**Authors:** Keijiro Sugimura, Hiroshi Miyata, Tomoyuki Matsunaga, Kei Asukai, Yoshitomo Yanagimoto, Yusuke Takahashi, Akira Tomokuni, Kazuyoshi Yamamoto, Akita Hirofumi, Junichi Nishimura, Masaaki Motoori, Hiroshi Wada, Hidenori Takahashi, Masayoshi Yasui, Takeshi Omori, Masayuki Ohue, Masahiko Yano

**Affiliations:** ^1^ Department of Digestive Surgery Osaka International Cancer Institute Osaka Japan

**Keywords:** anastomotic stenosis, cervical esophagogastric anastomosis, esophageal cancer, modified Collard anastomosis, propensity score matching

## Abstract

**Background:**

Several studies have reported that modified Collard anastomosis is useful for cervical anastomosis after esophagectomy for thoracic esophageal cancer. However, no large‐scale study has confirmed the efficacy of the modified Collard anastomosis.

**Methods:**

Between 2008 and 2016, 398 consecutive esophageal cancer patients who underwent esophagectomy and cervical anastomosis were enrolled in this study. Patients with a short remnant cervical esophagus were excluded. We investigated the utility of the modified Collard anastomosis by comparing the results of postoperative complications using a propensity score‐matched analysis between the hand‐sewn method (HS) and the modified Collard anastomosis (MC) for esophagogastric anastomosis of the neck after esophagectomy in thoracic esophageal cancer patients.

**Results:**

Of the 398 patients, 127 were included in the MC group and 127 were included in the HS group after propensity score matching. Clinical characteristics did not differ between the two groups. Frequency of anastomotic leakage tended to be lower in the MC group than in the HS group (3% vs. 7%, *P *=* *0.127). Frequency of anastomotic stenosis was significantly lower in the MC group than in the HS group (13% vs. 59%, *P *<* *0.001). Multivariate logic analysis showed that anastomotic technique (HS) and performance status were independent factors associated with anastomotic stenosis (odds ratio, 12.24 and 2.52; *P*‐value <0.001 and 0.047, respectively).

**Conclusion:**

In cervical esophagogastric anastomosis after esophagectomy, the modified Collard anastomosis is more suitable than hand‐sewn anastomosis in terms of reducing the frequency of anastomotic stenosis.

## 1 INTRODUCTION

Radical esophagectomy provides the best cure for patients with resectable esophageal cancer. However, this represents the most invasive procedure, and the frequency of postoperative complications is reported to range from 45% to 80%.^1^, ^2^, ^3^ Of the postoperative complications, anastomotic complications, such as anastomotic leakage and stenosis, are among the major concerns.

The anastomotic technique after esophagectomy has been investigated extensively in terms of location (intrathoracic or neck), suturing (hand‐sewn or mechanical) and type (end‐to‐end, side‐to‐side, or end‐to‐side).^4^, ^5^ However, the optimal anastomotic method has not yet been established. Hand‐sewn anastomosis is widely used, and leakage rate and stenosis rate for this approach are 0%‐33% and 2%‐89%, respectively.^4^, ^5^

Side‐to‐side anastomosis using a linear stapler was reported by Collard et al^6^ and modified by Orringer et al.^7^ This modified Collard method is considered to have made progress in reducing anastomotic complications. According to published reports, esophagogastric anastomosis using the modified Collard method has low rates of anastomotic leakage and stenosis.^8^, ^9^ In our hospital, in principle, hand‐sewn anastomosis was carried out for cervical esophagogastric anastomosis after esophagectomy until 2011. After 2012, the modified Collard anastomosis has been used. Few reports have compared these methods with other anastomotic methods, and such reports included small numbers of cases.

In the present large‐scale study, we investigated the utility of the modified Collard anastomosis by comparing data regarding postoperative complications between the hand‐sewn method and the modified Collard anastomosis in the esophagogastric anastomosis of the neck after esophagectomy in thoracic esophageal cancer patients.

## 2 METHODS

### 2.1 Patients

**Figure 1:**
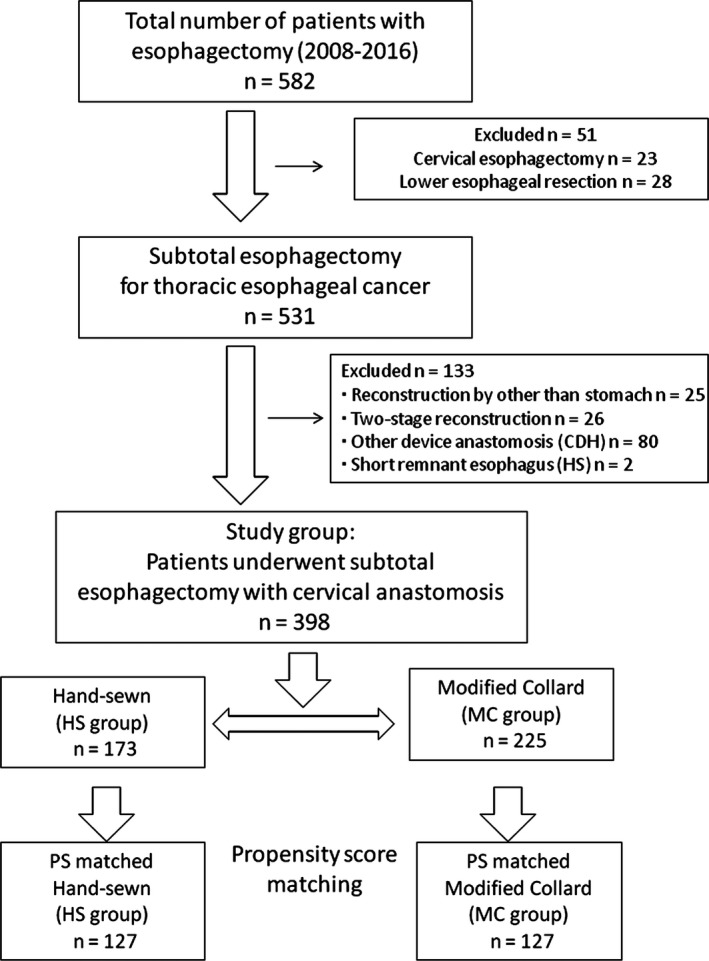
Patient selection for the evaluation of cervical esophagogastric anastomosis after esophagectomy in patients with thoracic esophageal cancer. CDH, curved intraluminal stapler (Ethicon)

Between January 2008 and December 2016, 582 consecutive patients with esophageal cancer underwent esophagectomy with radical lymph node dissection at the Osaka International Cancer Institute in Japan. Of these patients, 531 with thoracic esophageal cancer underwent subtotal esophagectomy and cervical esophagogastric anastomosis. Of those 531 patients, 25 underwent reconstruction using the jejunum or colon, 26 underwent two‐stage reconstruction, 80 underwent esophagogastric anastomosis using a circular stapling technique and two underwent hand‐sewn anastomosis because the remnant cervical esophagus was too short to undergo the modified Collard method. After excluding the above 133 patients, 398 patients were enrolled in the present study (Figure 1). The 7th edition of the Union for International Cancer Control TNM staging system was used. Details of preoperative treatment in our institution were described priviously.^10^, ^11^ The Human Ethics Review Committee of the Osaka International Cancer Institute approved the protocol of this retrospective cohort study (No. 1609089101).

### 2.2 Surgical procedure

Patients underwent esophagectomy and extensive mediastinal lymph node dissection through a right thoracotomy in the left‐lateral decubitus position. Patients were subsequently repositioned in the supine position, and cervical and abdominal lymph node dissection were then carried out. Cervical lymph node dissection was not carried out in lower thoracic esophageal cancer patients without cervical or upper mediastinal lymph node metastasis. Abdominal lymph node dissection was carried out using either open laparotomy or hand‐assisted laparoscopic surgery. A 4‐cm‐wide gastric conduit was created using a linear stapler along the greater curvature of the stomach. At the point of the reconstruction route, in our institution, the retrosternal route is routinely adopted. When the retrosternal route was unable to be used, the posterior mediastinal or percutaneous route was used. After the gastric conduit was pulled up to the neck, esophagogastric anastomosis was carried out on the left side of the neck. Regardless of any reconstruction route, anastomosis was done in the same way. In the present study, four surgeons carried out each method of anastomosis. We divided the four surgeons into two groups; two senior surgeons with over 20 years experience and two junior surgeons with under 20 years experience.

The hand‐sewn anastomosis was carried out as follows. First, an appropriate site was selected on the anterior wall of the gastric tube away from the stapled line approximately 2 cm below the highest point of the gastric tube to ensure good blood flow. Then, interrupted posterior seromuscular sutures were made using 3‐0 vicryl to approximate the esophagus and stomach. The stomach was then opened transversely approximately 3‐5 mm away from the posterior seromuscular suture line. Interrupted stitches through the full thickness of the stomach and esophagus were placed to achieve mucosa‐to‐mucosa approximation. The anterior wall of the anastomosis was completed in a way similar to that of the posterior wall.

**Figure 2:**
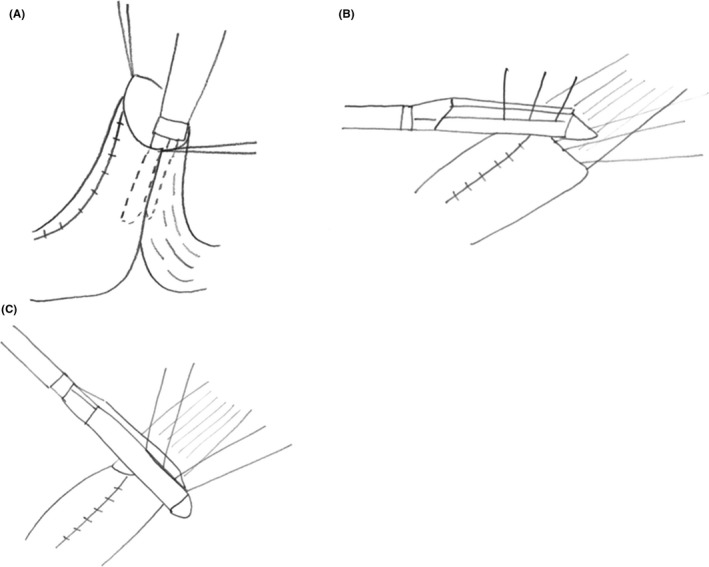
Modified Collard anastomosis. A, A linear stapler is applied to construct the posterior wall of the anastomosis. B,C, The anterior wall is closed using the linear stapler twice

The modified Collard anastomosis was carried out as follows (Figure 2). The cervical esophagus was approximately mobilized and delivered through the cervical incision together with the gastric tube. The tip of the gastric tube was resected 3‐5 cm from the top as a result of poor blood flow. The stay suture was placed on the posterior wall of the esophagus and gastric tube. A linear cutting stapler (ATS 45; Ethicon, Somerville, NJ, USA) was used to construct the posterior wall on the anastomosis. Then, after the stay suture of the anterior wall was done, the anterior wall was closed using the linear stapler twice. The seromuscular suture of the anterior wall was added, and the anastomosis was replaced orthotopically.

Regarding the criteria of anastomotic methods of our institution, in patients with a long remnant esophagus, cervical hand‐sewn anastomosis was carried out until December 2011. After January 2012, the modified Collard anastomosis has been used as an institutional change in patients with a long remnant esophagus. However, in patients with a short remnant esophagus, circular stapler or hand‐sewn anastomosis has been used for cervical anastomosis. In the present study, patients with a short remnant esophagus were excluded (Figure 1).

### 2.3 Perioperative management

Postoperative management was identical for both groups. After the operation, patients were admitted to the intensive care unit under anesthesia. On the day after surgery, the patients were extubated. Jejunostomy feeding was started after extubation. A nasogastric tube was used routinely and was suctioned every 2 hours. The tube was removed on postoperative day (POD) 5. Videofluorography was carried out on POD 8, and we then evaluated swallowing function and the state of the anastomotic site. Oral intake was started on POD 9 when the above two points were satisfactory. After discharge from the hospital, the patients were observed at the outpatient clinic every 2‐3 weeks. Three months after the surgery, regular blood tests and diagnostic imaging were carried out every 3 months until at least 5 years or recurrence.

### 2.4 Definition of perioperative complications

In the present study, anastomotic leakage was defined as the presence of saliva leaking through the cervical wound or the presence of extraluminal contrast as seen by videofluorography/computed tomography or visualization of dehiscence or fistula by endoscopy. Anastomotic stricture was defined according to previous methods.^12^ When the patient complained of dysphagia after surgery, endoscopic examination was carried out. The anastomotic site was observed using an XQ260 or XQ240 fiberscope with a front‐edge size of 9.0 mm (Olympus, Tokyo, Japan). If we were not able to push through the anastomotic site, presence of anastomotic stricture was defined. If the patient did not complain of dysphagia until 1 year after surgery, routine endoscopic examination was carried out 1 year after surgery, and the presence or absence of stricture was diagnosed. Dilation frequency was defined as the number of times endoscopic balloon dilatation was carried out until an endoscopic dilatation‐free state was achieved for at least 3 months after the last dilatation. Reflux esophagitis was defined as greater than grade A according to the Los Angeles Classification System. Concerning other postoperative morbidities such as pneumonia and recurrent nerve paralysis, ≥grade 2 postoperative morbidities according to the Clavien‐Dindo classification were defined as the appearance of complications.

### 2.5 Propensity matched analysis

Propensity matched analysis was conducted using a logistic regression model and the following covariates: age, gender, performance status (PS), concomitant disease, tumor location, clinical T factor, cN factor, cM factor, c stage, preoperative chemotherapy, preoperative chemoradiotherapy, route of reconstruction, field of dissection, and approach taken in the thoracic procedure.

### 2.6 Statistical analysis

Continuous variables are expressed as means ± SD. The χ^2^ test or Fisher's exact test was used to compare categorized variables. The Wilcoxon test was used to compare continuous variables. The Mann‐Whitney *U* test was used to compare sequential variables. The Cox proportional regression model was used to analyze univariate and multivariate factors. All calculations were carried out using the JMP v9.0.1 software program (SAS Institute Inc., Cary, NC, USA), and *P* values <0.05 were considered significant.

## 3 RESULTS

### 3.1 Patients and characteristics

Of the 398 patients, 173 patients were included in the hand‐sewn group (HS group), and the remaining 225 were included in the modified Collard group (MC group) before matching. After matching, 127 patients were included in the HS group, and 127 were included in the MC group (Figure 1). Table 1 shows the clinical characteristics of the patients before and after matching. After matching, there was no significant difference in backgrounds between the two groups.

**Table 1:** Characteristics of thoracic esophageal cancer patients

	Before matching			After matching		
	HS group (n = 173)	MC group (n = 225)	*P* value	HS group (n = 127)	MC group (n = 127)	*P* value
Age (y)						
Mean (range)	63 (37‐80)	67 (37‐80)	0.001	64 (42‐79)	66 (42‐80)	0.084
Gender						
Male	138 (80%)	180 (80%)	1.000	102 (80%)	106 (83%)	0.625
Female	35 (20%)	45 (20%)		25 (20%)	21 (17%)	
BMI (kg/m^2^)	21.0 ± 2.8	21.5 ± 3.1	0.130	20.9 ± 2.7	21.4 ± 3.1	0.192
Tobacco						
Yes	150 (87%)	196 (87%)	1.000	114 (90%)	115 (91%)	1.000
No	23 (13%)	29 (13%)		13 (10%)	12 (9%)	
Alcohol						
Yes	146 (84%)	206 (92%)	0.025	108 (85%)	116 (91%)	0.113
No	27 (16%)	19 (8%)		19 (15%)	11 (9%)	
Location						
Ut	22 (13%)	19 (8%)	0.368	11 (9%)	15 (12%)	0.576
Mt	98 (57%)	131 (58%)		74 (58%)	76 (60%)	
Lt	53 (30%)	75 (34%)		42 (33%)	36 (28%)	
Histological type						
SCC	167 (97%)	214 (95%)	0.209	124 (97%)	122 (96%)	0.365
Adenocarcinoma	6 (3%)	7 (3%)		3 (3%)	3 (3%)	
Others	0 (0%)	4 (2%)		0 (0%)	2 (1%)	
PS						
0	142 (82%)	193 (86%)	0.007	110 (87%)	108 (85%)	0.858
1	31 (18%)	24 (11%)		17 (13%)	19 (15%)	
2	0 (0%)	8 (3%)		0 (0%)	0 (0%)	
Concomitant disease						
Yes	81 (47%)	141 (63%)	0.002	65 (51%)	73 (57%)	0.378
No	92 (53%)	84 (37%)		62 (49%)	54 (43%)	
cT						
1	48 (28%)	74 (33%)	0.427	37 (29%)	33 (26%)	0.615
2	36 (21%)	41 (18%)		27 (21%)	27 (21%)	
3	71 (41%)	88 (39%)		49 (39%)	53 (42%)	
4	18 (10%)	22 (10%)		14 (11%)	14 (11%)	
cN						
0	77 (44%)	108 (48%)	0.504	60 (47%)	52 (41%)	0.326
1	69 (40%)	84 (37%)		48 (38%)	53 (42%)	
2	24 (14%)	31 (14%)		17 (13%)	20 (15%)	
3	3 (2%)	2 (1%)		2 (2%)	2 (2%)	
cM						
0	156 (90%)	208 (93%)	0.363	116 (91%)	114 (90%)	0.831
1	17 (10%)	16 (7%)		11 (9%)	13 (10%)	
cStage						
1	55 (32%)	89 (40%)	0.269	47 (37%)	41 (32%)	0.326
2	40 (23%)	36 (16%)		24 (19%)	22 (17%)	
3	60 (35%)	84 (37%)		45 (35%)	51 (40%)	
4	18 (10%)	16 (7%)		11 (9%)	13 (10%)	
Preoperative therapy						
Chemotherapy	73 (42%)	110 (49%)	0.394	13 (10%)	15 (12%)	0.733
Chemoradiotherapy	20 (46%)	90 (40%)		59 (46%)	63 (50%)	
None	80 (20%)	25 (11%)		55 (43%)	49 (39%)	

BMI, body mass index; HS, hand sewn method; MC, modified Collard anastomosis; PS, performance status; SCC, squamous cell carcinoma.

### 3.2 Operative outcome

Operative outcome for each group is summarized in Table 2. There was no significant difference in surgical experience of the surgeons between the two groups. Before matching, the operative time was significantly shorter in the MC group than in the HS group (523 minutes vs. 551 minutes, *P* = 0.003). The total blood loss was significantly less in the MC group than in the HS group (625 mL vs. 1074 mL, *P* < 0.001). After matching, the tendency was the same. The frequency of blood transfusion was similar between the two groups (39.9% vs. 32.0%, *P* = 0.113).

**Table 2:** Outcomes of thoracic esophageal cancer patients after surgery

	Before matching			After matching		
	HS group (n = 173)	MC group (n = 225)	*P* value	HS group (n = 127)	MC group (n = 127)	*P* value
Thoracic approach						
Right thoracotomy	167 (97%)	157 (70%)	<0.001	121 (95%)	121 (95%)	1.000
Thoracoscopic	6 (3%)	68 (30%)		6 (5%)	6 (5%)	
Abdominal approach						
Open	172 (99%)	89 (40%)	<0.001	126 (99%)	55 (43%)	<0.001
Hand‐assisted	1 (1%)	136 (60%)		1 (1%)	72 (57%)	
Dissection						
Two‐field	57 (33%)	89 (40%)	0.174	45 (35%)	40 (31%)	0.595
Three‐field	116 (67%)	136 (60%)		82 (65%)	87 (69%)	
Route of reconstruction						
Retrosternal	158 (91%)	200 (89%)	0.310	118 (93%)	116 (91%)	0.888
Posterior mediastinal	11 (6%)	13 (6%)		6 (5%)	7 (6%)	
Subcutaneous	4 (3%)	12 (5%)		3 (2%)	4 (3%)	
Surgeons						
Junior	85 (49%)	110 (49%)	1.000	62 (49%)	61 (48%)	1.000
Senior	88 (51%)	115 (51%)		65 (51%)	66 (52%)	
Total operative time (min)	551 ± 75	523 ± 65	0.003	545 ± 71	522 ± 67	0.015
Blood loss (mL)	1074 ± 702	625 ± 440	<0.001	1033 ± 487	690 ± 440	<0.001
Blood transfusion						
Yes	69 (40%)	72 (32%)	0.113	53 (42%)	45 (35%)	0.367
No	104 (60%)	153 (68%)		74 (58%)	82 (65%)	

HS, hand sewn method; MC, modified Collard anastomosis.

### 3.3 Postoperative outcome

Table 3 shows the postoperative complications for the two groups. Anastomotic leakage was less frequent in the MC group than in the HS group, but the difference did not reach statistical significance (3% vs. 8%, *P* = 0.063). Anastomotic stenosis was significantly less frequent in the MC group than in the HS group (15% vs. 59%, *P* < 0.001). Furthermore, the period between esophagectomy and the first dilatation was significantly shorter in the HS group than in the MC group (60 days vs. 91 days, *P* = 0.001). Number of endoscopic balloon dilatations, which was measured from the beginning to the release of stricture, was significantly lower in the MC group than in the HS group (4 vs. 3, *P* = 0.017). After matching, the tendencies regarding anastomotic leakage and stenosis were similar. There was no significant difference in other complications between the two groups. Mortality rate was similar between the two groups (0.6% vs. 0.9%, *P* = 0.640). Postoperative hospital stay was shorter in the MS group than in the HS group (23 days vs. 32 days, *P* < 0.001).

**Table 3:** Postoperative complications and postoperative course of thoracic esophageal cancer patients

	Before matching			After matching		
	HS group (n = 173)	MC group (n = 225)	*P* value	HS group (n = 127)	MC group (n = 127)	*P* value
Anastomotic leakage	13 (8%)	7 (3%)	0.063	9 (7%)	4 (3%)	0.127
Anastomotic stricture	102 (59%)	22 (10%)	<0.001	75 (59%)	16 (13%)	<0.001
First dilatation (d)	60 (27‐346)	91 (13‐251)	<0.001	51 (27‐346)	91 (29‐251)	0.009
Frequency of dilatation (times)	4 (1‐19)	3 (1‐12)	0.017	5 (1‐19)	2 (1‐11)	0.033
Reflux esophagitis	7 (4%)	2 (1%)	0.045	5 (4%)	1 (1%)	0.213
Pneumonia	22 (13%)	22 (10%)	0.421	16 (13%)	13 (10%)	0.694
Vocal cord palsy	24 (14%)	19 (8%)	0.103	16 (13%)	11 (9%)	0.416
Pneumothorax	3 (2%)	1 (0%)	0.321	3 (2%)	0 (0%)	0.247
Chylothorax	3 (2%)	4 (2%)	0.756	2 (2%)	2 (2%)	1.000
Postoperative bleeding	6 (4%)	2 (1%)	0.083	2 (2%)	1 (1%)	1.000
Arrhythmia	11 (6%)	10 (4%)	0.499	9 (7%)	6 (5%)	0.596
Ileus	0 (0%)	2 (1%)	0.507	0 (0%)	0 (0%)	1.000
Others	11 (6%)	11 (5%)	0.660	9 (7%)	9 (7%)	1.000
Reoperation	9 (5%)	2 (1%)	0.012	6 (5%)	1 (1%)	0.066
Overall morbidity	135 (78%)	94 (42%)	<0.001	97 (76%)	54 (42%)	<0.001
Mortality	1 (0.6%)	2 (0.9%)	0.640	1 (1%)	1 (1%)	1.000
Postoperative hospital stay	32 (14‐334)	23 (12‐228)	<0.001	32 (14‐267)	23 (12‐228)	<0.001

HS, hand sewn method; MC, modified Collard anastomosis.

### 3.4 Multivariate logistic analysis of anastomotic stenosis

Finally, we carried out logistic analysis to identify the independent factors associated with anastomotic stenosis after esophagectomy (Table 4). Results showed that PS and anastomotic technique (HS) were independent factors associated with anastomotic stenosis (odds ratios, 2.52 and 12.24; *P*‐values, 0.047 and <0.001, respectively).

**Table 4:** Univariate and multivariate analysis for the anastomotic stenosis

	Univariate analysis			Multivariate analysis		
	Odds ratio	95% CI	*P* value	Odds ratio	95% CI	*P* value
Age (≧65 y)	1.11	0.66‐1.86	0.680	1.21	0.62‐2.36	0.578
Gender (male)	1.52	0.77‐3.16	0.230	1.85	0.76‐4.78	0.177
BMI (≧22 kg/m^2^)	1.22	0.72‐2.09	0.457	1.06	0.55‐2.09	0.847
Tobacco	1.49	0.62‐3.96	0.382	1.21	0.62‐2.36	0.578
Alcohol	1.11	0.48‐2.43	0.802	1.05	0.39‐2.88	0.919
Location (Lt)	2.20	1.27‐3.82	0.005	2.21	0.99‐5.02	0.052
PS (≧1)	1.74	0.85‐3.55	0.129	2.52	1.01‐6.35	0.047
Concomitant disease	1.11	0.66‐1.87	0.682	1.14	0.59‐2.22	0.691
cStage (≧3)	1.51	0.90‐2.55	0.116	1.69	0.87‐3.33	0.122
Preoperative chemoradiotherapy (none)	3.76	1.39‐13.09	0.007	3.08	0.91‐12.8	0.072
Two‐field dissection	1.41	0.82‐2.41	0.209	1.07	0.49‐2.37	0.858
Anastomosis (hand‐sewn)	10.00	5.43‐19.36	<0.001	12.24	6.27‐25.40	<0.001
Anastomotic leakage	2.18	0.70‐6.97	0.174	1.43	0.38‐5.42	0.590

BMI, body mass index; PS, performance status.

## 4 DISCUSSION

In the present study, we evaluated the utility of the modified Collard anastomosis and compared it with that of hand‐sewn anastomosis using propensity score‐matched analysis in cervical esophagogastric anastomosis after esophagectomy. Results showed that the frequency of anastomotic leakage was similar between the two groups. The results also showed that anastomotic stenosis was significantly less frequent in the MC group than in the HS group. These results show that modified Collard anastomosis is effective in reducing the incidence of anastomotic stenosis. This is the first report comparing treatment outcomes between the modified Collard anastomosis and other methods of anastomosis in a large‐scale study.

The results of the present study showed that anastomotic leakage was less frequent in the MC group than in the HS group, but this difference was not statistically significant. Compared with the 13.3% frequency in the Japanese nationwide database, anastomotic leakage was less frequent in both groups of our study.^13^ According to Honda's review of 12 prospective randomized controlled trials, the frequency of anastomotic leakage in hand‐sewn anastomosis was 6%,^5^ similar to our results for the HS group. Deng et al^4^ reviewed 15 prospective randomized controlled trials and retrospective studies. The frequency of leakage in these trials was 18%, which was higher than our results for the HS group, although this previous study included patients with cervical anastomosis only. In contrast, Collard et al^6^ first reported cervical side‐to‐side anastomosis using a linear stapler and reported an anastomotic leakage incidence of 6%. Ercan et al^8^ also reported on modified Collard anastomosis and noted that the incidence of anastomotic leakage was 4%. Prince et al^14^ also reported on the modified Collard anastomosis and noted that the incidence of anastomotic leakage was 21%. Compared to these previous reports, the incidence of leakage in our large‐scale study was lower. According to our results, modified Collard anastomosis advantageously shows a lower rate of anastomotic leakage.

The results of the present study showed that anastomotic stenosis was significantly less frequent in the MC group than in the HS group. In this study, the stenosis rate in the HS group was 59%. According to the review by Deng et al,^4^ the stenosis rate of hand‐sewn anastomoses was 54%, similar to our results. According to another review by Honda et al,^5^ the stenosis rate of hand‐sewn anastomoses was 10%, lower than our results. The cause of these differences is unknown, although one explanation for the high incidence of anastomotic stenosis in the surgical procedures used might be the small size of the anastomotic site and the use of a double‐layer suture in both the anterior and posterior walls. Another explanation for these differences is that the resection length in the tip of the gastric conduit was too short in the HS group. In the HS group, ischemia around the anastomotic site might result in anastomotic stenosis. In contrast, according to previous reports regarding the Collard anastomosis, the rate of anastomotic stenosis ranged widely from 4.6% to 66%. Ercan et al^8^ reported a frequency of anastomotic stenosis of 66%, higher than that in our study. Prince et al^14^ reported a frequency of stenosis of 24%, whereas Behzadi et al^9^ reported a frequency of anastomotic stenosis of 4.6%, lower than that in our study. Probable explanations for this difference are that the follow‐up period was short and that anastomotic stenosis was defined as being restricted to patients with dysphagia who underwent endoscopic balloon dilatation. Another possible explanation for this difference is the insertion length of the staple in the anastomosis of the posterior wall. One of the advantages of the modified Collard anastomosis is its large anastomotic diameter. Behzadi et al reported that a 45‐50‐mm insertion length of the staple in the anastomosis resulted in a low stenosis rate. Logically, the longer the staple that is inserted, the larger the anastomosis diameter that can be formed. As a result, the stenosis rate can be decreased. However, if the insertion length is increased, the blood flow at the top of the gastric conduit may become worse. In this study, our insertion length was 40 mm. Thus, further studies should investigate the appropriate insertion length. According to the results of the present study, the modified Collard anastomosis advantageously reduces the incidence of anastomotic leakage.

In our group, the first choice for the reconstruction route was the retrosternal route. If the retrosternal route reconstruction could not be carried out, a posterior mediastinal route or a subcutaneous route was chosen. In the MC group of the present study, the leakage rate was 0% in both posterior mediastinal and subcutaneous reconstructions, and the stenosis rates were 25% and 8%, respectively (Table S1). In the posterior mediastinal or subcutaneous route reconstruction, the modified Collard anastomosis might also be useful as well as in the retrosternal route. One explanation as to why anastomotic leakage happened only in the retrosternal route in the MC group is that the number of patients with posterior mediastinal or subcutaneous route reconstruction was small. Another explanation is that the sternoclavicular joint may impinge on the retrosternal conduit. It is possible that gastric pull‐up by the retrosternal route may cause more mechanical stress to the stomach than the other routes, resulting in reduced perfusion, and oxygen supply. It might eventually lead to anastomotic leakage in the retrosternal reconstruction.

In the present study, after matching, a significant difference still exists in total operative time and blood loss between the two groups. The cause of the difference between the two groups may be attributed to the difference in abdominal approach or learning curve associated with the surgical period. The difference in surgical invasiveness between the two groups may result in the different frequencies in anastomotic stenosis. Indeed, according to an analysis of postoperative anastomotic stenosis by Ahmed et al,^15^ anastomotic stenosis was associated with intraoperative hypoperfusion.

In the current study, poor PS was an independent factor associated with anastomotic stenosis. We investigated whether poor PS was associated with specific concomitant diseases. Results showed that poor PS tended to be associated with the presence of cardiovascular disease (*P* = 0.097). According to the analysis of anastomotic stenosis by Ahmed et al,^15^ ASA grade and cardiovascular disease were independent risk factors for anastomotic stenosis. Our results were consistent with their findings.

The present study had limitations. This was a retrospective consecutive cohort study. From now, we should prospectively investigate the efficacy of the modified Collard anastomosis.

In conclusion, in cervical esophagogastric anastomosis after esophagectomy, the modified Collard anastomosis is superior to hand‐sewn anastomosis in terms of reducing the frequency of anastomotic stenosis. To validate the above results, prospective clinical trials should be carried out.

## DISCLOSURE

Funding: The authors declare no sources of support.

Conflicts of interest: Authors declare no conflicts of interest for this article.

## References

[1] Putnam JB Jr , Suell DM , McMurtrey MJ , et al. Comparison of three techniques of esophagectomy within a residency training program. Ann Thorac Surg. 1994;57:319–25. 8311591 10.1016/0003-4975(94)90990-3

[2] McCulloch P , Ward J , Tekkis PP . Mortality and morbidity in gastro‐oesophageal cancer surgery: initial results of ASCOT multicentre prospective cohort study. BMJ 2003;327:1192–7. 14630753 10.1136/bmj.327.7425.1192 PMC274052

[3] Ando N , Kato H , Igaki H , et al. A randomized trial comparing postoperative adjuvant chemotherapy with cisplatin and 5‐fluorouracil versus preoperative chemotherapy for localized advanced squamous cell carcinoma of the thoracic esophagus (JCOG9907). Ann Surg Oncol. 2011;19:68–74. 21879261 10.1245/s10434-011-2049-9

[4] Deng XF , Liu QX , Zhou D , Min JX , Dai JG . Hand‐sewn vs linearly stapled esophagogastric anastomosis for esophageal cancer: a meta‐analysis. World J Gastroenterol. 2015;21:4757–64. 25914488 10.3748/wjg.v21.i15.4757 PMC4402326

[5] Honda M , Kuriyama A , Noma H , Nunobe S , Furukawa TA . Hand‐sewn versus mechanical esophagogastric anastomosis after esophagectomy: a systematic review and meta‐analysis. Ann Surg. 2013;257:238–48. 23001084 10.1097/SLA.0b013e31826d4723

[6] Collard JM , Romagnoli R , Goncette L , Otte JB , Kestens PJ . Terminalized semimechanical side‐to‐side suture technique for cervical esophagogastrostomy. Ann Thorac Surg. 1998;65:814–7. 9527220 10.1016/s0003-4975(97)01384-2

[7] Orringer MB , Marshall B , Iannettoni MD . Eliminating the cervical esophagogastric anastomotic leak with a side‐to‐side stapled anastomosis. J Thorac Cardiovasc Surg. 2000;119:277–88. 10649203 10.1016/S0022-5223(00)70183-8

[8] Ercan S , Rice TW , Murthy SC , Rybicki LA , Blackstone EH . Does esophagogastric anastomotic technique influence the outcome of patients with esophageal cancer? J Thorac Cardiovasc Surg. 2005;129:623–31. 15746747 10.1016/j.jtcvs.2004.08.024

[9] Behzadi A , Nichols FC , Cassivi SD , Deschamps C , Allen MS , Pairolero PC . Esophagogastrectomy: the influence of stapled versus hand‐sewn anastomosis on outcome. J Gastrointest Surg. 2005;9:1031–40. 16269373 10.1016/j.gassur.2005.06.025

[10] Yamasaki M , Yasuda T , Yano M , et al. Multicenter randomized phase II study of cisplatin and fluorouracil plus docetaxel (DCF) compared with cisplatin and fluorouracil plus Adriamycin (ACF) as preoperative chemotherapy for resectable esophageal squamous cell carcinoma (OGSG1003). Ann Oncol. 2017;28:116–20. 27687307 10.1093/annonc/mdw439

[11] Sugimura K , Miyata H , Yano M , et al. Is 18F‐FDG‐PET useful for predicting R0 resection after induction therapy for initially unresectable locally advanced esophageal carcinoma? Gen Thorac Cardiovasc Surg. 2017;65:455–62. 28585161 10.1007/s11748-017-0786-9

[12] Sugimua K , Motoori M , Yano M , et al. Endoscopic steroid injection reduced frequency of repeat dilation in patients with anastomotic stenosis after esophagectomy. Esophagus. 2016;13:62–7.

[13] Takeuchi H , Miyata H , Gotoh M , et al. A risk model for esophagectomy using data of 5354 patients included in a Japanese nationwide web‐based database. Ann Surg. 2014;260:259–66. 24743609 10.1097/SLA.0000000000000644

[14] Price TN , Nichols FC , Harmsen WS , et al. A comprehensive review of anastomotic technique in 432 esophagectomies. Ann Thorac Surg. 2013;95:1154–60. 23395626 10.1016/j.athoracsur.2012.11.045

[15] Ahmed Z , Elliott J , King S , et al. Risk factors for anastomotic stricture post‐esophagectomy with a standardized sutured anastomosis. World J Surg. 2017;41:487–97. 27778075 10.1007/s00268-016-3746-0

