# Supplementary material for: Comparison of the modified Collard and hand‐sewn anastomosis for cervical esophagogastric anastomosis after esophagectomy in esophageal cancer patients: A propensity score‐matched analysis
Source: Ann Gastroenterol Surg. 2018 Nov 8;3(1):104–13. doi: 10.1002/ags3.12220 (PMC6345657; doi:10.1002/ags3.12220)
Supplement: Supplementary file 1 [file AGS3-3-104-s001.docx]

|  | Before matching | |  |  | After matching | |  |
| --- | --- | --- | --- | --- | --- | --- | --- |
|  | HS group  (n=173) | MC group  (n=225) | p-value |  | HS group  (n=127) | MC group  (n=127) | p-value |
| Anastomotic leakage |  |  |  |  |  |  |  |
| retrosternal | 10/158 (6%) | 7/200 (4%) | 0.023 |  | 7/118 (6%) | 4/116 (3%) | 0.539 |
| posterior mediastinal | 1/11 (9%) | 0/13 (0%) | 0.458 |  | 0/6 (0%) | 0/7 (0%) | 1.000 |
| subcutaneous | 2/4 (50%) | 0/12 (0%) | 0.050 |  | 2/3 (67%) | 0/4 (0%) | 0.143 |
|  |  |  |  |  |  |  |  |
| Anastomotic stenosis |  |  |  |  |  |  |  |
| retrosternal | 96/158 (61%) | 29/200 (15%) | <0.001 |  | 9/118 (59%) | 15/116 (13%) | <0.001 |
| posterior mediastinal | 3/11 (27%) | 1/13 (8%) | 0.142 |  | 2/6 (33%) | 0/7 (0%) | 0.192 |
| subcutaneous | 3/4 (75%) | 3/12 (25%) | 0.118 |  | 3/3 (100%) | 1/4 (25%) | 0.143 |

Supplementary Table1

Frequency of anastomotic leakage and stenosis by reconstruction route
